# Development of Chrysin Loaded Oil-in-Water Nanoemulsion for Improving Bioaccessibility

**DOI:** 10.3390/foods10081912

**Published:** 2021-08-18

**Authors:** Pisamai Ting, Wanwisa Srinuanchai, Uthaiwan Suttisansanee, Siriporn Tuntipopipat, Somsri Charoenkiatkul, Kemika Praengam, Boonrat Chantong, Piya Temviriyanukul, Onanong Nuchuchua

**Affiliations:** 1 Doctoral Program in Nutrition, Faculty of Medicine Ramathibodi Hospital and Institute of Nutrition, Mahidol University, Bangkok 10400, Thailand; pisamai.tig@student.mahidol.ac.th; 2 Nano Agricultural Chemistry and Processing Research Team, National Nanotechnology Center (NANOTEC), National Science and Technology Development Agency (NSTDA), Klong Luang, Pathum Thani 12120, Thailand; wanwisa.sri@nanotec.or.th; 3 Institute of Nutrition, Mahidol University, Salaya, Phuttamonthon, Nakhon Pathom 73170, Thailand; uthaiwan.sut@mahidol.ac.th (U.S.); siriporn.tun@mahidol.ac.th (S.T.); somsri.chr@mahidol.ac.th (S.C.); kemika.pra@mahidol.ac.th (K.P.); 4 Department of Pre-Clinical and Applied Animal Science, Faculty of Veterinary Science, Mahidol University, Salaya, Phutthamonthon, Nakhon Pathom 73170, Thailand; boonrat.cha@mahidol.edu

**Keywords:** bioaccessibility, chrysin, encapsulation, flavonoid, nanoemulsion

## Abstract

Chrysin (5,7-dihydroxyflavone) is a remarkable flavonoid exhibiting many health-promoting activities, such as antioxidant, anti-inflammatory, and anti-Alzheimer’s disease (AD). Nevertheless, chrysin has been addressed regarding its limited applications, due to low bioaccessibility. Therefore, to improve chrysin bioaccessibility, a colloidal delivery system involving nanoemulsion was developed as chrysin nanoemulsion (chrysin-NE) using an oil-in-water system. Our results show that chrysin can be loaded by approximately 174.21 µg/g nanoemulsion (100.29 ± 0.53% *w/w*) when medium chain triglyceride (MCT) oil was used as an oil phase. The nanocolloidal size, polydispersity index, and surface charge of chrysin-NE were approximately 161 nm, 0.21, and −32 mV, respectively. These properties were stable for at least five weeks at room temperature. Furthermore, in vitro chrysin bioactivities regarding antioxidant and anti-AD were maintained as pure chrysin, suggesting that multistep formulation could not affect chrysin properties. Interestingly, the developed chrysin-NE was more tolerant of gastrointestinal digestion and significantly absorbed by the human intestinal cells (Caco-2) than pure chrysin. These findings demonstrate that the encapsulation of chrysin using oil-in-water nanoemulsion could enhance the bioaccessibility of chrysin, which might be subsequently applied to food and nutraceutical industries.

## 1. Introduction

Flavonoids are polyphenolic secondary metabolites mostly found in fruits and vegetables. They can be divided into six subgroups based on hydroxylation and substitution as flavones, isoflavones, flavonols, flavanones, flavanols, and anthocyanidins [1]. Flavonoids are of interest in the functional food and nutraceutical industries because of their strong health-promoting effects on several ailments, including antidiabetes, antimutagenic, antihypertension, antiaging, and anti-Alzheimer’s disease [2,3].

Chrysin (5,7-dihydroxyflavone) is a natural flavonoid containing a 15-carbon flavone backbone and falls within the flavone subgroup with apigenin, baicalein, and luteolin. Chrysin is abundantly found in *Oroxylum indicum* (L.) Kurz, *Passiflora caerulea*, *Passiflora edulis*, and *Radix scutellaria*, as well as propolis [4,5]. Interestingly, all these plants exhibit medicinal properties [4,5]. Chrysin has been reported for its biological activities, including antioxidant, antiobesity, anti-inflammation, antidiabetes, and neuroprotective effects [4,5]. Administration of chrysin (20 mg/kg bodyweight) exerted antioxidant activities to prevent D-galactose-mediated aging in rats by maintaining tissue antioxidant enzyme activities [6]. The same dose of chrysin also reduced malondialdehyde (MDA), an oxidative stress biomarker, in both the liver and kidney of rats exposed to drinking ethanol [7]. Moreover, oral supplementation of a high concentration of chrysin (100 mg/kg body weight) decreased ammonium chloride-induced neuroinflammatory responses by suppressing the expression of pro-inflammatory cytokines associated with various chronic diseases [8]. However, whether chrysin possesses health benefits remains unclear, since it exhibits poor water-solubility properties at 20 µg/mL [9]. The disposition and metabolism of chrysin in healthy volunteers showed that ingested chrysin (400 mg) and its metabolite, chrysin sulfate, reached optimal contents within an hour after ingestion at 3–16 ng/mL and was excreted via feces at up to 98% in an unchanged form, implying extremely low bioavailability of chrysin at 0.003% [10]. These findings suggested that the alleviating effect of chrysin was limited because of its low oral bioaccessibility, mainly due to poor solubility, extensive metabolism, and efflux of metabolites back into the intestine for hydrolysis and fecal elimination [10,11,12]. Hence, this raises the key question concerning how the oral bioaccessibility of chrysin could be improved to enhance its health benefits.

In the last decade, nanocarrier technology has been used to entrap drugs or phytochemicals and reduce the bottleneck of chemical properties, such as instability, insolubility, or low bioaccessibility and bioavailability. In the food industry, colloidal nanoemulsion delivery systems are most commonly employed [13] compared to liposomes, solid lipid nanoparticles, and polymeric nanoparticles [14]. Polymeric nanoparticles have been applied for organic solvent impurities, large polymer aggregates, toxic monomers, and toxic degradation products [15], while liposomes, solid lipid nanoparticles are less toxic than polymeric nanoparticles; however, large-scale production is concerned.

Nanoemulsion was defined by McClements, 2010 as the formulation from two fluids that are immiscible with each other, with one of the fluids being dispersed as small spherical droplets in the other [16], and kinetically stabilized in water (droplet size range 20–500 nm) [17,18]; however, the upper limit for droplet size is not well-determined yet. Nanoemulsion has been focused on as an excellent carrier to improve the solubility and stability of lipophilic bioactive compounds by enhancing membrane diffusion that may promote bioavailability and therapeutic effects [17,18,19]. There have been reports on the formulation of chrysin-loaded nanoparticles with many additional excipients, the use of organic solvents, the time-consuming preparation process, and moderate entrapment efficiency [9,20,21]. Therefore, the low bioaccessibility of chrysin was resolved in this study by encapsulation in an oil-in-water nanoemulsion (chrysin-NE). The advantages of our formulation include the reduction of multiple steps of preparation and various additional excipients, as well as the elimination of organic solvents, resulting in a simple preparation with increased entrapment efficiency. The bioactivities regarding antioxidant activities and in vitro anti-Alzheimer’s disease (AD) properties between chrysin and chrysin-NE were performed to examine the effect of heat generated during the nanoemulsion process. Moreover, Chrysin-NE bioaccessibility in human intestinal cells (Caco-2) as a cell model for intestinal absorption and the epithelial barrier was also investigated. Our data highlighted the advantages of an oil-in-water nanoemulsion to improve chrysin entrapment efficiency, bioaccessibility and endorse future use in the food and nutraceutical industries.

## 2. Materials and Methods

### 2.1. Solubility of Chrysin in Oil Phase

Chrysin (2.5 mg, 98.9% Titration from Tokyo Chemical Technology, Tokyo, Japan) was dissolved in absolute ethanol (1 mL). Then, the chrysin/ethanol solution was added into different edible oils (including medium chain triglyceride (MCT) oil from Healtholicious One-Stop (Bangkok, Thailand), rice bran oil, sunflower seed oil, grape seed oil, and camellia seed oil, ranging from 0.04–1.74 mg chrysin per 1 g oil, until an immiscible phase appeared. The edible oil containing the highest content of chrysin was further used as an oil phase to develop nanoemulsions.

### 2.2. Development of Chrysin-Loaded Oil-in-Water Nanoemulsions

Chrysin-loaded oil-in-water nanoemulsions (chrysin-NE) were fabricated following the formulas shown in Table 1. The oil phase was composed of edible oil (7.5% *w/w*), ethanol (7.5% *w/w*), and sorbitan monooleate (Span 80; 1–2% *w/w*). The water phase consisted of polysorbate 20 (Tween 20; 1–2% *w/w*) and water (81–83% *w/w*). To make an emulsion, the water phase and the oil phase were heated at 60 °C with agitation. The two phases were mixed and homogenized by an IKA T 25 Digital ULTRA-TURRAX^®^ Disperser, Staufen, Germany, at a speed of 10,000 rpm for 3 min. Finally, the chrysin-NE was developed by passing the pre-emulsion through a microfluidizer (Microfluidizer^®^, LV1 Low Volume, ON, Canada). Pressurization was carried out at 15,000 psi for three cycles. Nanocolloids of chrysin-NE were then analyzed in terms of mean particle size, particle size distribution (polydispersity index; PdI), and surface charge (zeta potential) by a Zetasizer (Malvern Panalytical Technologies, Malvern, UK). To avoid multiple scattering effects, the samples were diluted in deionized water (DI) at a volume ratio of 1 to 100. All measurement was carried out in triplicate with the refractive index parameter at 1.33. Chrysin-NE was stored at 25 °C until required for further analysis.

### 2.3. Quantification of Chrysin Encapsulated in Nanoemulsions

The quantity of chrysin in chrysin-NE was measured using high-performance liquid chromatography (HPLC) coupled with a W2690/5 Autosampler, a 2695 Pump, and a 2998 Photodiode Array Detector (Waters, Santa Clara, CA, USA). Chrysin separation was performed by a Symmetry C18 HPLC Column (XSelect HSS C18 3.0 mm × 75 mm, 2.5 µm, Waters, Santa Clara, CA, USA) and an isocratic elution (60% *v/v* acetonitrile and 40% *v/v* water containing 1% *v/v* acetic acid) at a flow rate of 0.2 mL/min. Chrysin was detected by UV absorption at a wavelength of 270 nm [22]. The HPLC chromatograms were analyzed by Empower 2 software (Agilent, Santa Clara, CA, USA).

The quantity of chrysin in chrysin-NE was determined using the calibration curve generated from the peak areas of authentic chrysin (1–25 µg/mL) (Supplementary Figure S1). The HPLC validation parameters (e.g., linear regression equation, correlation coefficient (R^2^), limit of detection (LOD), limit of quantification (LOQ), precision, and accuracy) are shown in Supplementary Table S1. The encapsulation efficiency of chrysin in chrysin-NE was calculated as follows:

% Entrapment efficiency of chrysin = 100 × [C_NE_/C_int_], (1)

where C_int_ and C_NE_ are the concentrations of initial chrysin added in nanoemulsion and chrysin measured in chrysin-NE, respectively.

### 2.4. Determination of 2,2-Diphenyl-1-Picrylhydrazyl Scavenging Activity

To measure 2,2-diphenyl-1-picrylhydrazyl (DPPH) radical scavenging activity of chrysin and chrysin-NE, the reaction of 300 µL of chrysin and chrysin-NE (167 µg/mL) was diluted with 750 µL of dimethyl sulfoxide (DMSO) to destroy emulsion. DPPH radical scavenging activities were then determined following the method used in a previous report [23]. Trolox (160 ng/mL) was used as the positive control, and the radical scavenging activity was calculated as a percentage of DPPH discoloration using the following equation:

% DPPH scavenging activity = 100 × (1 − [Abs_sample_/Abs_control_]) (2)

where Abs_sample_ is the absorbance of the sample, and Abs_control_ is the absorbance of 95% (*v/v*) aqueous ethanol.

### 2.5. Determination of Cholinesterase Inhibitory Activities

Cholinesterases, including acetylcholinesterase (AChE) and butyrylcholinesterase (BChE) inhibitory activities, were performed as previously reported [24] with some modifications as follows. The assay consisted of 100 µL of 2 µg/mL *Electrophorus electricus* AChE (1000 units/mg) in 50 mM potassium phosphate buffer (KPB, pH 7.0), 50 μL of 2 mM acetylthiocholine, 10 µL of 16 mM 5,5′-dithiobis (2-nitrobenzoic acid) (DTNB) and 40 µL of sample. The sample was prediluted in DMSO to destroy the emulsion, as previously mentioned. For BChE inhibitory activities, the assay consisted of 100 µL of 0.5 µg/mL equine serum BChE (≥10 units/mg) in 50 mM KPB containing 1 mM MgCl_2_, 50 μL of 10 mM butyrylthiocholine, 10 µL of 16 mM DTNB and 40 µL of sample. All enzymes and chemicals were sourced from Sigma-Aldrich (St. Louis, MO, USA). The assay was measured at 412 nm using a 96-well microplate reader and Gen5 data analysis software (BioTek Instruments, Inc., Winooski, VT, USA). The percentage of enzyme inhibition was calculated according to the following equation.

(3) $$\%\;\mathrm{inhibition}\;=\;\left(1\;-\;\frac{B-b}{A-a}\right)\;\times \;100,$$

where *A* is the initial velocity of the reaction with enzyme, *a* is the initial velocity of the reaction without enzyme, *B* is the initial velocity of the enzyme reaction with the sample, and *b* is the initial velocity of the reaction with extract, but without enzyme.

### 2.6. In Vitro Digestion and Bioaccessibility

In vitro gastrointestinal tract (GIT) digestion of chrysin and chrysin-NE was conducted to track their fate within a gastric and intestinal phase according to a previous protocol [25,26]. Chrysin or chrysin-NE at 232 µg/mL was mixed with salt solution (120 mM NaCl, 6 mM CaCl_2_, and 5 mM KCl), with pH decreased to 2.0 ± 0.1 to represent gastric digestion with pepsin (924 units/mg protein) and a final concentration of pepsin at 2 mg/mL. The sample was incubated in a shaking water bath (Memmert, Schwabach, Germany) for an hour at 37 °C. To simulate small intestinal digestion, 0.1 M NaHCO_3_ was added to raise the pH to 6.0 ± 0.2, and porcine bile extract (containing glycine and taurine conjugates of hyodeoxycholic acid and other bile salts), pancreatin (a combination of trypsin, amylase and lipase, ribonuclease, and protease; activity equivalent to 4× United States Pharmacopoeia (U.S.P.) specification) and lipase solution (100–650 units/mg protein) were added and incubated in a shaking water bath for another two hours at 37 °C. Final concentrations of bile extract, pancreatin, and lipase were 2.4, 0.4, and 0.2 mg/mL, respectively. All enzymes were sourced from Sigma-Aldrich (St. Louis, MO, USA). After completing the simulated small intestinal phase of digestion, the sample was centrifuged at 6797× *g* for 45 min at room temperature to isolate the aqueous fraction. After centrifugation, the supernatant was filtered through a 0.22 µm polytetrafluoroethylene (PTFE) filter to obtain the fraction with mixed micelles. The micellar fraction of chrysin-NE was subjected to particle size and surface charge characterizations, as previously mentioned.

### 2.7. Cellular Uptake and Transport of Chrysin

Differentiated monolayers of human intestinal-like Caco-2 cells (HTB-37, American Type Culture Collection, Rockville, MD, USA) were used to investigate the uptake and transepithelial flux of chrysin. Details for the growth, maintenance, and experimental use of these cells were previously described [27]. To perform the experiments, differentiated cultures of Caco-2 cells on transmembrane inserts (21–25 days after reaching confluency) were used at passages 24 and 33. The fraction with mixed micelles was diluted 1/4 with Dulbecco’s minimal essential medium (DMEM) (pH 6.5), and denoted as the test medium. The monolayers were washed once with basal DMEM before the test medium was added to the apical compartment. The basolateral medium consisted of phenol red-free DMEM. Cultures were incubated at 37 °C in a humidified atmosphere. After 4 h, apical, basolateral, and cell samples were collected and extracted by trichloroacetic acid (1.5 mg/mL) in acetonitrile [28]. The cytotoxicity of the test medium towards Caco-2 cells was also evaluated by MTT assay and reported as a percentage of cell viability compared to control digestion (mixture of digestive enzymes without chrysin).

To determine chrysin and chrysin-NE levels, chromatographic separation was performed on an Agilent 6495 Triple Quad LC/MS (Santa Clara, CA, USA) with a positive ion scan mode, electrospray ionization (ESI), and a Poroshell 120 SB-C18 column (2.1 mm × 100 mm, 2.7 µm) (Agilent, Santa Clara, CA, USA). Gradient elution was applied using type I water containing 0.1% (*v/v*) formic acid (mobile phase A) and acetonitrile containing 0.1% (*v/v*) formic acid (mobile phase B). The gradient program consisted of 100% A at 0–1 min with flow rate 0.2 mL/min, 50% A at 1–10 min with flow rate 0.2 mL/min, 10% A at 11–17 min with flow rate 0.05 mL/min, 10% A at 17–22 min with flow rate 0.10 mL/min and 100% A at 22–25 min with flow rate 0.2 mL/min. The positive ion mass (*m/z*) of chrysin was 255.24 [M + H]^+^. Quantitation was performed based on the full scan analysis and extracted ion chromatograms using Mass Hunter Software (Agilent, Santa Clara, CA, USA). Bioaccessibility was defined as the amount of chrysin that was partitioned in the filtered aqueous fraction during simulated digestion to become available for uptake and possible transport across small intestinal absorptive epithelial cells and calculated using the following equation.

% Bioaccessibility of chrysin = 100 × [C_chry_/C_int_], (4)

where, C_int_ and C_chry_ are the concentrations of chrysin in the initial nanoemulsion and digestion during GIT model, respectively.

### 2.8. Statistical Analysis

The experimental results were analyzed by one-way ANOVA with posthoc Tukey’s HSD test or Student’s *t*-test (SPSS Statistics version 18.0). Significance levels were determined using *p* values as indicated in the legends. All experiments were carried out in triplicate (*n* = 3), with data expressed as mean ± standard deviation (SD).

## 3. Results

### 3.1. Formulations and Characterizations of Chrysin Nanoemulsions (Chrysin-NE)

Chrysin is a lipophilic compound that is immiscible in oil. Ethanol was chosen as a co-solvent to dissolve chrysin in edible oils. Table 2 shows that chrysin could be loaded in MCT oil at the maximum capacity up to 1680.93 ± 54.15 µg/g, followed by rice bran oil (178.09 ± 5.74 µg/g), sunflower seed oil (84.78 ± 2.73 µg/g), grape seed oil (41.30 ± 1.33 µg/g) and camellia seed oil (40.90 ± 1.32 µg/g), respectively. Therefore, MCT oil was selected as the oil phase for developing chrysin-loaded oil-in-water nanoemulsions (chrysin-NE) in further experiments.

**Figure 1:**
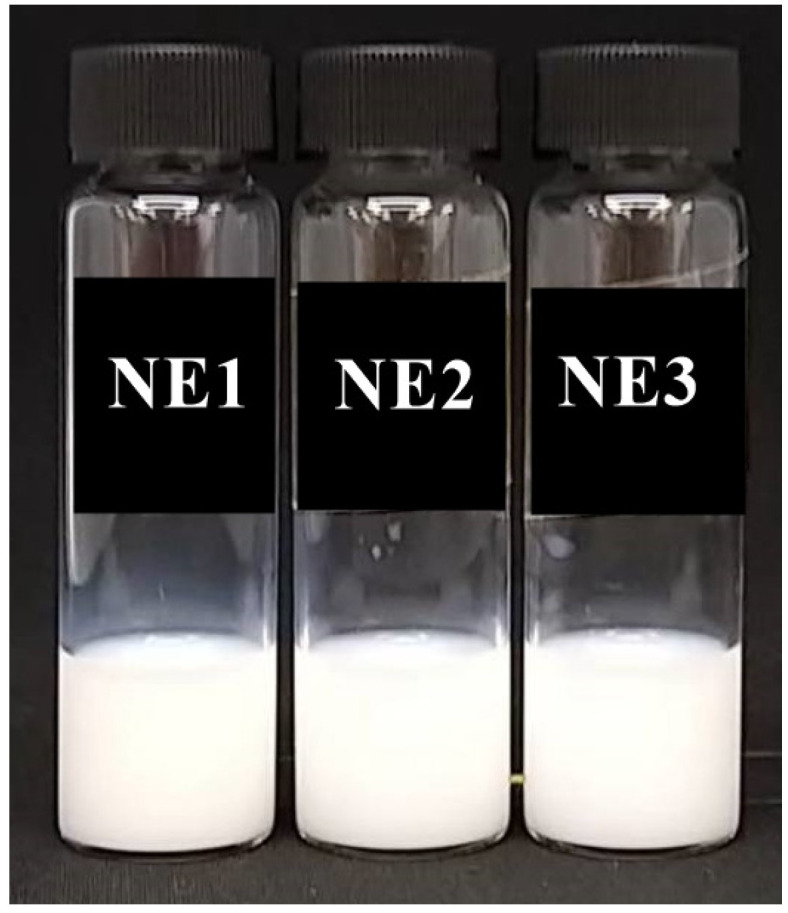
Chrysin nanoemulsion formulas NE1, NE2, and NE3 on the production day, pressurized at 15,000 psi for three cycles.

Chrysin nanocolloids obtained after the pre-emulsions were suspended by the microfluidization process for three cycles. Physical appearances of chrysin nanoemulsions (NE1, NE2, and NE3) are shown in Figure 1. On the production day, chrysin nanoemulsions ranged from 110 to 160 nm with a narrow size distribution (PdI = 0.21–0.23). The nanoparticles had negative charges between −22 and −32 mV. Results are illustrated in Table 3.

The chrysin NE1, NE2, and NE3 nanoemulsion formulas were then characterized regarding mean particle size, polydispersity index (PdI), and zeta potential surface charge (Table 3). Results showed that 15% *w/w* oil phase (containing 7.5% *w/w* ethanol and 7.5% *w/w* MCT oil) could be loaded in nanoemulsions and stabilized by 2–4% *w/w* of a co-surfactant system containing polysorbate 20 (Tween 20) and sorbitan monooleate (Span 80) at a weight ratio of 1:1. However, only NE1 was chosen for the stability studies because NE2 and NE3 showed toxicity toward Caco-2 cells (data not shown), possibly because of the use of polysorbate 20 with concentrations higher than 1% *w/w* [29].

The chrysin-NE formula 1 (NE1) was stable after storage at 25 °C for five weeks. However, the nanoemulsion showed phase separation after three weeks when stored at 45 °C. Increases in nanocolloidal size and the polydispersity index, together with a change in particle charges, indicated the formation of non-uniform nanoparticles (data not shown). The stability study suggested that chrysin-NE stored at 25 °C was stabilized in the nanoemulsion during the experimental period. Total loaded chrysin was 174.21 ± 1.98 µg/g, whereas encapsulation efficiency of chrysin in chrysin-NE was 100.29 ± 0.53% *w/w* (Table 4).

### 3.2. Effects of Nanoemulsion Preparation on Chrysin Biological Activities

Whether the biological activities of chrysin remained because of the multistep formulation of chrysin-NE when the heat was generated was unclear. Therefore, we preliminary evaluated chrysin biological activities using in vitro assays. It has been previously reported that chrysin exhibited antioxidant activities by quenching 2,2-diphenyl-1-picrylhydrazyl (DPPH) radicals [30], and inhibited acetylcholinesterase (AChE) and butyrylcholinesterase (BChE), the enzymes involved in Alzheimer’s disease (AD) pathogenesis and cognitive decline [31]. The DPPH radical scavenging assay is a rapid antioxidant assay that can be used to determine the antioxidant capacity of plant extracts or food components [32]. The assay determines the antioxidant properties of tested compounds by acting as free radical scavengers against stable free radicals, DPPH. AChE and BChE are enzymes involved in the degradation of acetylcholine, which is one of the hallmarks of AD patients. Thus, AChE and BChE inhibition are drug targets for AD [31]. Together, DPPH radical scavenging activities and AD-related enzyme inhibitory activities of pure chrysin were analyzed and compared to chrysin-NE. Chrysin-NE was solubilized by DMSO to destroy the emulsion and release chrysin. Then, biological activities were evaluated using spectrophotometric methods.

**Figure 2:**
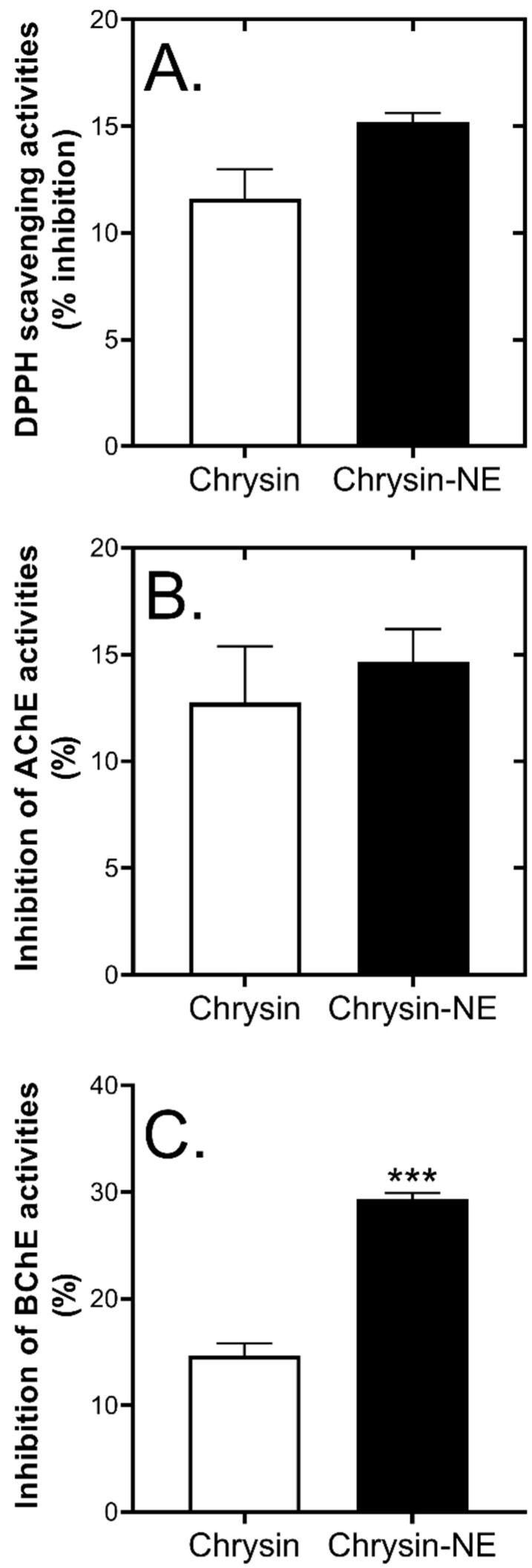
Effects of nanoemulsion on chrysin biological activities. (**A**) percentage inhibition of DPPH scavenging activity with a final concentration of chrysin at 46.5 µg/mL (**B**) percentage inhibition of AChE activity, and (**C**) percentage inhibition of BChE activity. Final concentration of chrysin in (**B**,**C**) was 9.5 µg/mL. Statistical significance was analyzed by Student’s *t*-test against pure chrysin. ***, *p* ≤ 0.001.

Figure 2A shows that pure chrysin quenched DPPH radicals at 11.62 ± 1.37%, similar to solubilized chrysin-NE at 15.20 ± 0.43%, and suggesting that chrysin was stable after multistep formulation. Chrysin also showed inhibitory activities against AChE and BChE. As shown in Figure 2B, pure chrysin (final concentration 9.5 µg/mL) inhibited AChE by 12.00 ± 2.65%, while solubilized chrysin-NE carrying the same amount of chrysin exhibited similar AChE inhibitory activities. Interestingly, Figure 2C displays that solubilized chrysin-NE inhibited BChE more than pure chrysin. It could be possible that medium chain fatty acids and chrysin may have a combined inhibitory effect on BChE [33]. Data showed that chrysin exhibited biological activities in vitro, even after nanoemulsion development.

### 3.3. Characteristics of Chrysin-NE during In Vitro Gastrointestinal Tract Digestion

Bioaccessibility relates to the fraction of a compound releasing from the food matrix in the oral digestive system, thereby made available for intestinal absorption [34,35]. Following encapsulation of chrysin, the fate of chrysin-NE in the gastrointestinal tract has been determined by in vitro digestion and bioaccessibility on human intestinal-like Caco-2 cells.

**Figure 3:**
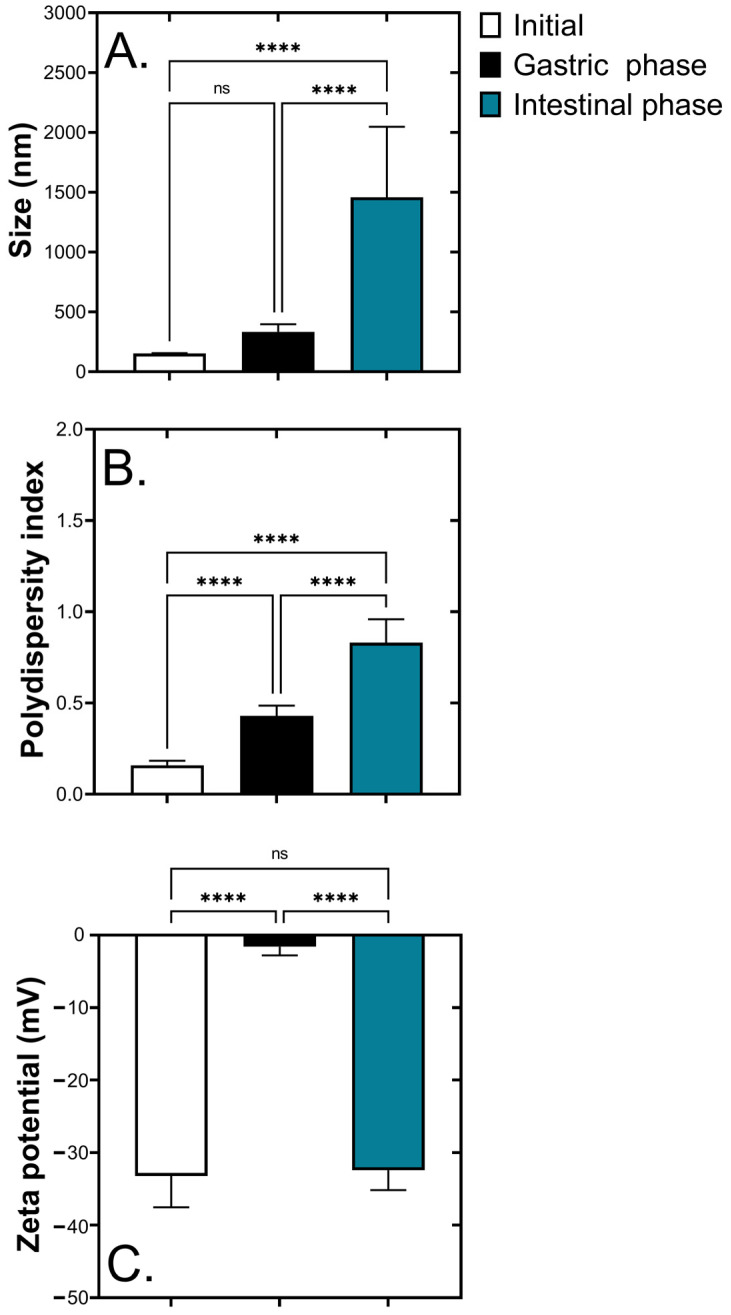
Effects of simulated gastrointestinal tract digestion on physical characteristics of chrysin nanoemulsion (chrysin-NE). (**A**) mean particle size in nm (**B**) polydispersity index, and (**C**) zeta potential (mV) of the chrysin-NE at the initial, gastric, and intestinal phases. Statistical analyses were determined by one-way ANOVA with posthoc Tukey’s HSD test. ****, *p* ≤ 0.001, ns = not significant.

After digestion, the characteristics of chrysin-NE, including mean particle size, polydispersity index, and zeta potential, were evaluated (Figure 3). Figure 3A shows that particle size measured by dynamic light scattering significantly increased from the initial stage to the stomach and intestinal phases at 152 ± 30, 334 ± 63, and 1457 ± 590 nm, respectively. The polydispersity index reflecting the particle size distribution of chrysin-NE also significantly increased from 0.16 ± 0.03 at the initial state to 0.83 ± 0.13 at the intestine (Figure 3B). Intriguingly, the electrical charge of chrysin-NE representing the zeta potential (Figure 3C) became less negative in the gastric phase (−1.60 ± 1.20 mV) compared with the initial state (−33.32 ± 4.32 mV), while the zeta potential was reversed to −32.44 ± 2.75 mV at the intestinal condition, implying strong acid conditions in the gastric phase.

### 3.4. Bioaccessibility of Chrysin and Chrysin-NE during In Vitro Gastrointestinal Tract Digestion

**Figure 4:**
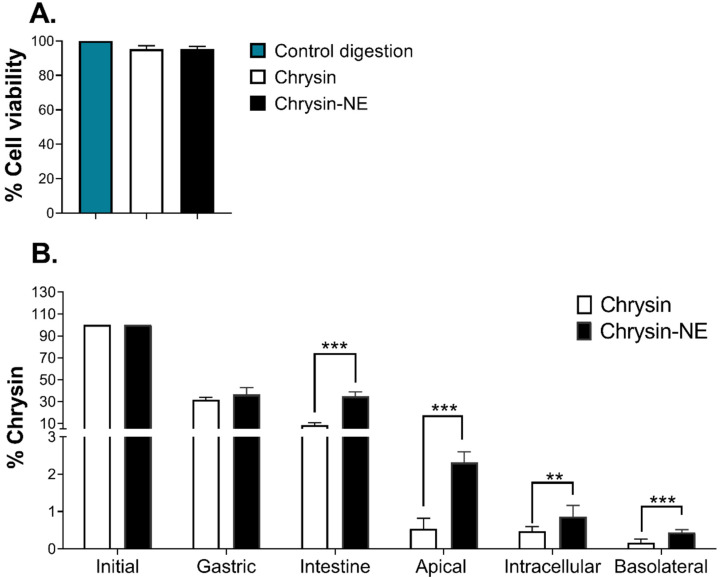
(**A**) Cytotoxicity analysis of the bioaccessible fraction of chrysin and chrysin-NE towards Caco-2 cells. (**B**) Effects of chrysin and chrysin-NE on bioaccessibility and absorption within the gastrointestinal tract using human intestinal-like Caco-2 cells. Percentages of remaining chrysin between pure chrysin and chrysin-NE at each step were compared. Statistical analysis was determined by Student’s *t*-test against pure chrysin at the indicated step. **, *p* ≤ 0.01, and ***, *p* ≤ 0.001.

To evaluate the bioaccessibility of chrysin and chrysin-NE, we first determined the cytotoxicity of the bioaccessible fraction, referred to the materials and methods section as the test medium. After intestinal digestion, the results indicated that chrysin and chrysin-NE were not toxic to Caco-2 cells when compared to the control digestion (mixtures of digestive enzymes, but without chrysin) (Figure 4A).

The fate of chrysin at each step of simulated gastrointestinal digestion was subsequently determined as a percentage (Figure 4B). At the initial step, the amount of chrysin and chrysin-NE was equal at 232 µg/mL. Later, chrysin-NE was digested at about 70% in the gastric phase, similar to pure chrysin. Notably, chrysin-NE was protected from intestinal digestion, while non-encapsulated chrysin was degraded 4-folds compared to chrysin-NE (Figure 4). The concentration of chrysin-NE was 4 to 5-folds higher than chrysin at the apical region of Caco-2 cells (% chrysin of chrysin-NE at the apical region was 2.31 ± 0.29%, while % chrysin of pure chrysin at the same region was 0.54 ± 0.29%), indicating enhanced opportunity of chrysin exposure of chrysin-NE. The intracellular and basolateral fractions also showed that chrysin-NE was significantly transported into the cells and released into the basolateral region at 2 to 3-folds higher than chrysin. Results showed that the nanoemulsion system significantly improved chrysin bioaccessibility by protecting chrysin against digestion at the intestinal step and enhanced intestinal absorption.

## 4. Discussion

An emulsion is a biphasic system of lipid and aqueous phases. To improve the water solubility of lipophilic compounds, oil-in-water systems are developed by dispersing an oil phase (containing bioactive compounds) in an aqueous phase using surfactants [17,36]. Colloidal particles can be formed at different submicron sizes (between 20 and 500 nm). These are called nanoemulsions [37] that can improve dispersibility and bioavailability of nutrient delivery systems, due to their kinetic stability, high surface-active area, and stabilizing property of active compounds [17,19,38]. Thus, oil-in-water nanoemulsions are applicable for oral delivery of lipophilic nutraceuticals, such as triterpenes [39,40], flavonoids, flavones [41,42], flavonols [43,44], and flavanones [45,46].

For chrysin, a natural flavonoid, MCT oil can be used as a carrier to develop a chrysin oil-in-water nanoemulsion. Chrysin solubility in MCT oil is higher than in other edible oils. Our results were similar to curcumin, a lipophilic flavonoid found in *Curcuma longa*. Curcumin can be dissolved in MCT oil at up to 2.02 mg/g compared to rapeseed oil, soybean oil, corn oil, and olive oil [47]. MCT oil developed a high curcumin-loaded nanoemulsion with high solubility of curcumin in MCT oil compared to coconut oil, olive oil, and corn oil [48].

After the chrysin-NE1 formulation, the water solubility of chrysin increased to 160 µg/g compared to the parent form at 20 µg/g [9]. The average size of chrysin nanocolloids ranged from 160 nm to 170 nm, while the distribution of the chrysin nanoparticles was homogenous, indicated by the low polydispersity index (0.21 to 0.27) (Table 3). The surface charge of the colloidal particles was close to the absolute value of 30 mV, suggesting a balance of adhesion/repulsion forces between the nanoparticles [18]. Chrysin-NE is stable when stored at 25 °C, and can be encapsulated in nanoemulsion for up to five weeks (Table 4). The hydrophilic-lipophilic balance (HLB) value for the use of Tween 20 and Span 80 in the nanoemulsion was equivalent to 10.25, resulting in high stability of the nanocolloids [49,50]. Furthermore, the encapsulation efficiency of chrysin in the nanoemulsion was 100.29 ± 0.53% *w/w*. Our results were similar to the encapsulation of chrysin in sodium oleate-based nanoemulsion (90% *w/w*) [9], in solid lipid nanoparticles (86% *w/w*) [51], and in chrysin-loaded poly(lactic-co-glycolic acid) nanoparticles (93% *w/w*) [52].

After the formulation and characterization of chrysin-NE, its bioaccessibility was evaluated by simulating gastrointestinal tract digestion and intestinal absorption. During digestion, physical alterations of chrysin-NE occurred. Particle size increased and became polydispersed in a wide range, correlating to morphological assessment by confocal microscopy. Figure 3 shows that during gastric digestion, change in the electrical surface charge from negative to nearly positive indicated that pepsin and acidic condition altered the ionic strength, including the electrical barrier of the nanoemulsion. This resulted in the particles merging (coalescence) and a mixed micelle formation similar to previous studies [34,35,53]. The presence of ions (Ca^2+^, K^+^, and H^+^) may also reduce the negative charge contents determined by zeta potentials [50]. Later, at the intestinal condition (pH = 6 ± 0.2), the electrical charge of chrysin-NE returned to a negative value, as in the initial stage. This zeta potential change was also observed in β-carotene nanoemulsion [54,55], and was explained by the presence of neutral pH, bile salts, and lipase [54]. Figure 4 shows that chrysin-NE was at least 4-folds more tolerant than pure chrysin (control) in digestion, leading to a high chance of exposure to intestinal cells. Chrysin-NE was protected during intestinal digestion by (i) merging into mixed micelles containing bile salts, phospholipids, and lipid digestion products [47], or (ii) a similar electrostatic repulsion at the initial and intestinal stages that protected chrysin-NE.

Finally, the digested chrysin-NE and chrysin (control) were evaluated for their bioaccessibility using the Caco-2 model. The majority content of chrysin was retained on the apical side of the Caco-2 cells compared to the control (Figure 4), implying prolonged sustainability of chrysin on the surface of the intestinal cells. The intestinal cells significantly uptook chrysin that appeared on the basolateral side as at least 2-folds higher than the control. The low bioavailability of chrysin may result from the efflux reaction of P-glycoprotein, and multidrug resistance-associated protein 2 (MRP2) expressed on the apical compartment of Caco-2 cells [56,57]. Enrichment of chrysin by nanoemulsion may compete with the efflux reaction. Interestingly, co-encapsulation of chrysin and quercetin, a flavonoid possessing MRP2 inhibiting activities, may increase chrysin absorption by promoting a transcellular mechanism [58].

Bioaccessibility is the first step in improving oral bioavailability [53,59]. Our findings highlighted that encapsulation of chrysin using an oil-in-water nanoemulsion was an appropriate delivery system to enhance bioaccessibility. The most recent literature on nanoemulsions suggests that their application is safe [60]. Application of chrysin-loaded oil-in-water nanoemulsions shows promise in several lines of daily food products, including fortified water fruit juices, coffee, soft drinks, creamy beverages, or salad dressings. The basic chrysin-NE could be developed in a powdered form by lyophilization or spray drying to enhance products in the food or supplement industries.

## Figures and Tables

**Table 1:** Compositions of oil and water phases in chrysin-loaded oil-in-water nanoemulsions and their particle characteristics on production day and after storing for five weeks at 25 °C.

Formulations	Oil Phase (% *w/w*)			Water Phase (% *w/w*)	
	MCT Oil	EtOH ^a^	Span 80	Tween 20	Water
Chrysin-NE 1	7.5	7.5	1	1	83
Chrysin-NE 2	7.5	7.5	1.5	1.5	82
Chrysin-NE 3	7.5	7.5	2	2	81

^a^ Containing 2.5 mg/mL chrysin; chrysin-NE, chrysin-nanoemulsion; MCT, medium chain triglyceride; span 80, sorbitan monooleate; tween 20, polysorbate 20.

**Table 2:** Solubilities of chrysin in edible oils.

Edible Oils	Chrysin Content (µg/g)
MCT	1680.93 ± 54.15
Rice bran	178.09 ± 5.74
Sunflower seed	84.78 ± 2.73
Grape seed	41.30 ± 1.33
Camellia seed	40.90 ± 1.32

**Table 3:** Particle characteristics of three formulas of chrysin-NE on the production day and after storage at 25 °C for five weeks.

Formulations	Size (nm)		PdI		Zeta Potential (mV)	
	Production Day	5 Weeks	Production Day	5 Weeks	Production Day	5 Weeks
Chrysin-NE 1	161 ± 1.96	173 ± 3.11	0.21 ± 0.01	0.27 ± 0.01	−32 ± 0.61	−30 ± 2.06
Chrysin-NE 2	122 ± 1.50	n/d	0.23 ± 0.01	n/d	−22 ± 0.51	n/d
Chrysin-NE 3	110 ± 0.40	n/d	0.20 ± 0.02	n/d	−23 ± 2.76	n/d

PdI, polydispersity index; n/d, no data.

**Table 4:** Content and entrapment efficacy of chrysin in nanoemulsion (NE1) stored at 25 °C for five weeks.

Chrysin Nanoemulsions	Chrysin Content (µg/g)	Entrapment Efficiency (% *w/w*)
Production day	174.21 ± 1.98	100.29 ± 0.53
1 week	174.37 ± 0.91	99.19 ± 0.68
3 weeks	171.28 ± 1.25	98.52 ± 0.72
5 weeks	179.54 ± 0.71	101.75 ± 2.76

## Data Availability

Data is contained within this article and supplementary materials.

## Supplementary Materials

The following are available online at https://www.mdpi.com/article/10.3390/foods10081912/s1, Table S1: The validation parameters of chrysin detection using the HPLC technique. Figure S1: High-performance liquid chromatograms of (A) chrysin, and (B) chrysin in nanoemulsion (NE1). Figure S2: Mass spectra of the deprotonated molecular ion of (A) chrysin, (B) chrysin in the gastric phase, (C) intestinal phase, (D) apical side of Caco-2 cells, (E) intracellular of Caco-2 cells, and (F) basolateral side of Caco-2 cells.
