# Supplementary material for: Development of Chrysin Loaded Oil-in-Water Nanoemulsion for Improving Bioaccessibility
Source: Foods. 2021 Aug 18;10(8):1912. doi: 10.3390/foods10081912 (PMC8392734; doi:10.3390/foods10081912)

foods

Supplementary materials

Development of Chrysin Loaded Oil-in-Water Nanoemulsion for Improving Bioaccessibility

Pisamai Ting [1], Wanwisa Srinuanchai [2], Uthaiwan Suttisansanee [3], Siriporn Tuntipopipat [3], Somsri Charoenkiatkul [3], Kemika Praengam [3], Boonrat Chantong [4], Piya Temviriyanukul [3,*,†] and Onanong Nuchuchua [2,*,†]

1 Doctoral Program in Nutrition, Faculty of Medicine Ramathibodi Hospital and Institute of Nutrition, Mahidol University, Bangkok 10400, Thailand
2 Nano Agricultural Chemistry and Processing Research Team, National Nanotechnology Center (NANOTEC), National Science and Technology Development Agency (NSTDA), Klong Luang, Pathum Thani 12120, Thailand
3 Institute of Nutrition, Mahidol University, Salaya, Phuttamonthon, Nakhon Pathom 73170, Thailand
4 Department of Pre-Clinical and Applied Animal Science, Faculty of Veterinary Science, Mahidol University, Salaya, Phutthamonthon, Nakhon Pathom 73170
* Correspondence: piya.tem@mahidol.ac.th (P.T.); onanong@nanotec.or.th (O.N.); Tel.: +66-2800-2380 (ext. 326) (P.T.); +66-2117-6792 (O.N.)
† These authors contributed equally to this work.

Supplementary Table S1:

The validation parameters of chrysin detection using HPLC technique.

Linear range (µg/mL)	Linear regression equation	Correlation coefficient (R^2)	LOD (µg/mL)	LOQ (µg/mL)	%RSD					
					Intraday			Interday		
					1 µg/mL	10 µg/mL	25 µg/mL	1 µg/mL	10 µg/mL	25 µg/mL
1-25	y = 345036x-49719	0.9999	0.0307	0.1023	0.88	1.15	1.76	15.78	7.86	5.53

Supplementary Figure S1:

High performance liquid chromatograms of (A.) chrysin, and (B.) chrysin in nanoemulsion (NE1)

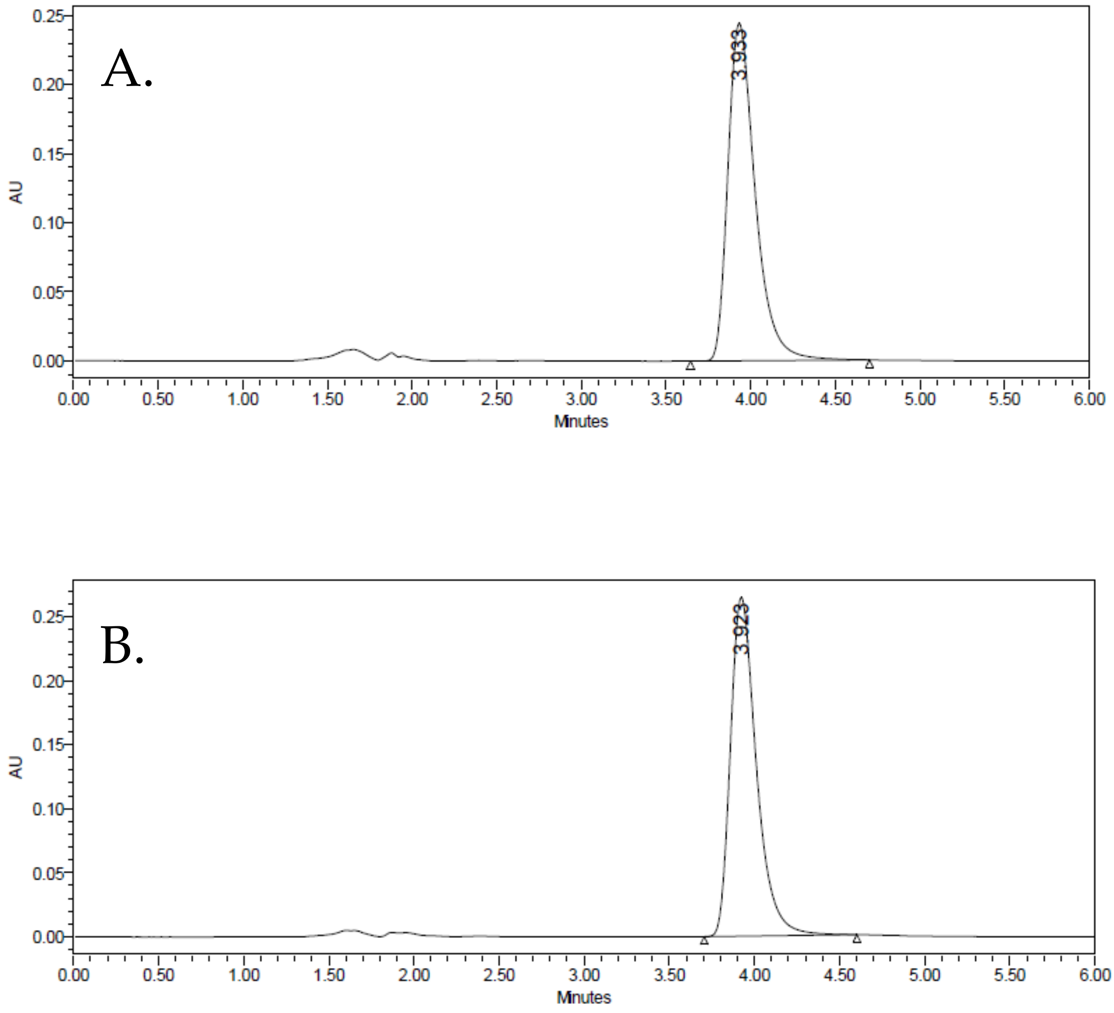

Supplementary Figure S2:

Mass spectra of deprotonated molecular ion of (A.) chrysin, (B.) chrysin in the gastric phase, (C.) intestinal phase, (D.) apical side of Caco-2 cells, (E.) intracellular of Caco-2 cells, and (F.) basolateral side of Caco-2 cells.

A.

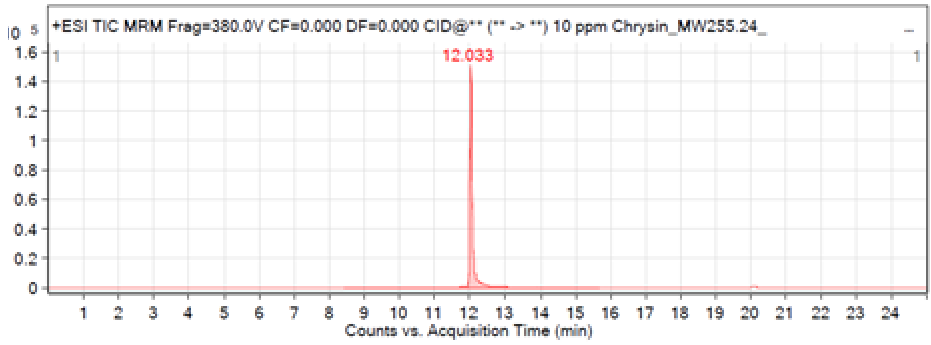

B.

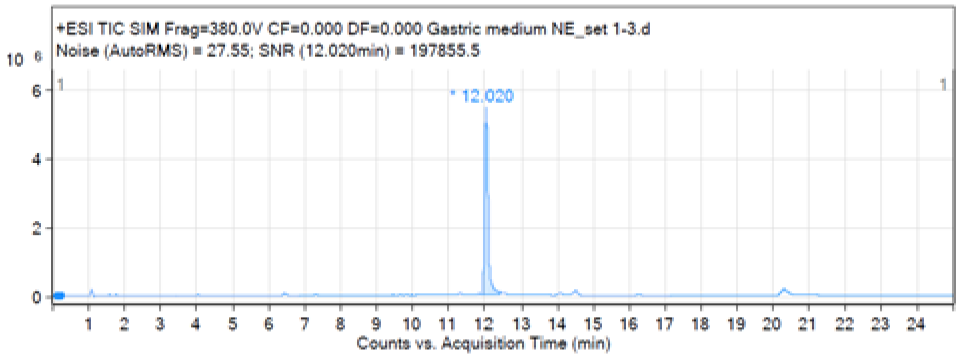

C.

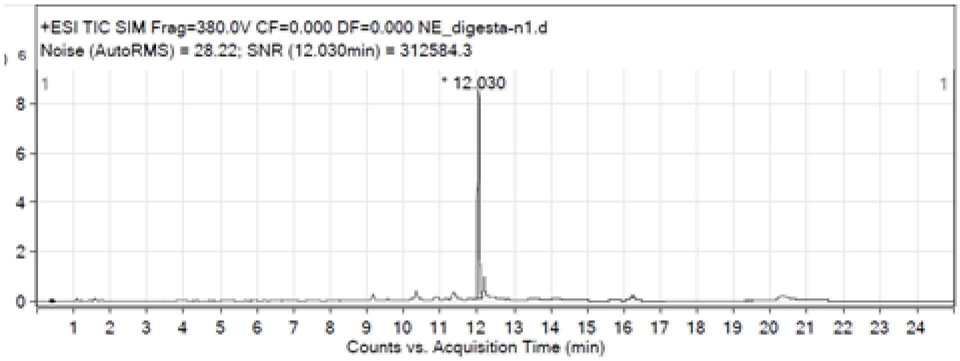

Supplementary Figure S2 (Cont.):

Mass spectra of deprotonated molecular ion of (A.) chrysin, (B.) chrysin in the gastric phase, (C.) intestinal phase, (D.) apical side of Caco-2 cells, (E.) intracellular of Caco-2 cells, and (F.) basolateral side of Caco-2 cells.

D.

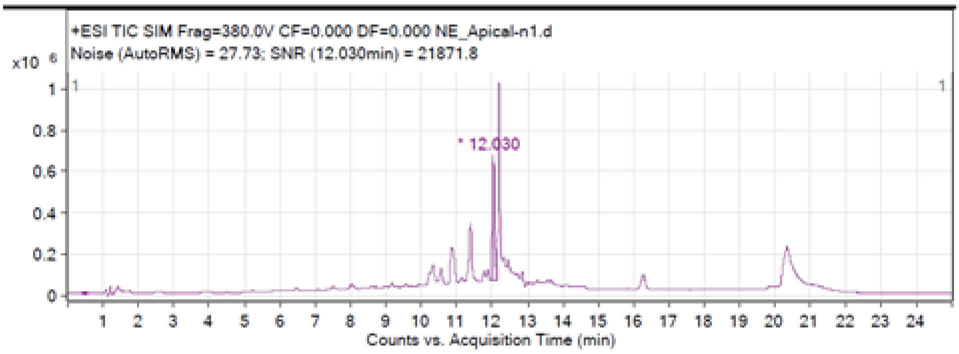

E.

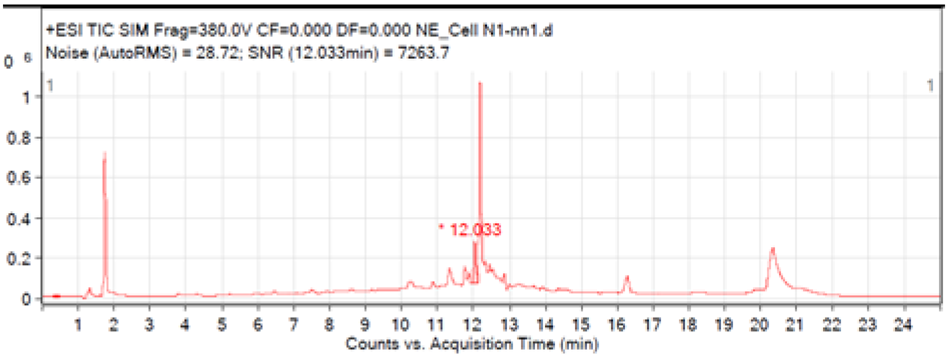

F.

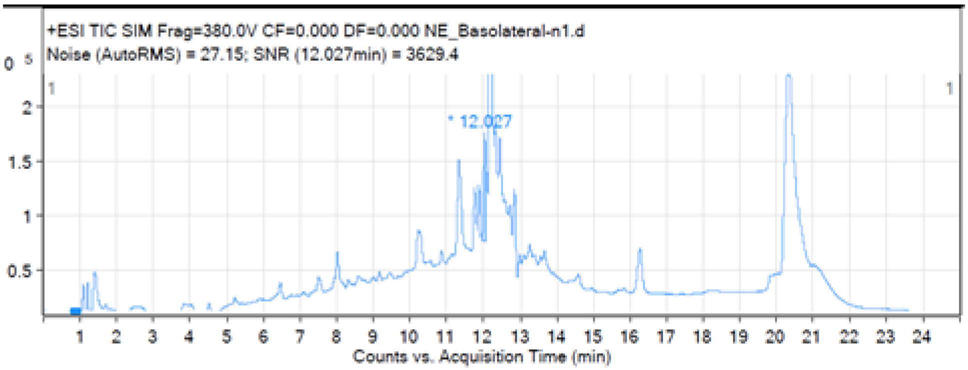

Supplement: Supplementary file 1 [file foods-10-01912-s001.zip › foods-1328304-supplementary.pdf]
